# Cutaneous and Gastrointestinal Symptoms in Two Patients with Systemic Mastocytosis Successfully Treated with Omalizumab

**DOI:** 10.1155/2015/903541

**Published:** 2015-01-28

**Authors:** Sofie Lieberoth, Simon Francis Thomsen

**Affiliations:** Department of Dermatology, Bispebjerg Hospital, 2400 Copenhagen NV, Denmark

## Abstract

The pathogenesis of mastocytosis is not well defined and thus treatment remains challenging and remains on a palliative basis. We present two cases (a 48-year-old woman and a 57-year-old man) with indolent systemic mastocytosis in whom omalizumab (anti-IgE) reduced gastrointestinal and cutaneous symptoms significantly. This observation provides additional insight into the effects of omalizumab on systemic mastocytosis.

## 1. Introduction

Mastocytosis refers to a group of myeloproliferative disorders characterized by excessive proliferation and accumulation of mast cells in tissues. Whereas cutaneous mastocytosis (CM) is limited to the skin, systemic mastocytosis (SM) describes forms of mastocytosis in extracutaneous organs, with or without skin involvement. The majority of SM patients present with a point mutation in the KIT gene at the 816 position. Systemic mastocytosis comprises clinical variants ranging from indolent systemic mastocytosis to mast cell leukaemia and is commonly associated with anaphylaxis and symptoms including palpitations, flushing, cutaneous itch, nausea, and diarrhoea. The WHO diagnostic major criterion for SM includes multifocal, dense aggregates of mast cells (15 or more) detected in sections of bone marrow and confirmed by tryptase immunohistochemistry or other special stains, whereas minor criteria are as follows: (a) in biopsy section, more than 25% of the mast cells in the infiltrate have atypical morphology, or, of all the mast cells in the aspirate smear, more than 25% are immature or atypical; (b) mast cells coexpress CD117 with CD2 and/or CD25; (c) detection of KIT point mutation at codon 816 in bone marrow, blood, or other extracutaneous organs; (d) serum total tryptase persistently >20 ng/mL (not a valid criterion in cases of systemic mastocytosis with associated clonal hematologic non-mast-cell lineage disease). A diagnosis of SM may be rendered if one major plus one minor criterion or three minor criteria are fulfilled [1]. The pathogenesis of mastocytosis is not well defined and thus treatment remains challenging and on a palliative basis [2, 3].

Omalizumab is a humanized murine monoclonal antibody that conjugates free serum IgE and reduces binding to the high-affinity IgE receptor (FC*ε*RI) on mast cells and basophils, thereby stabilizing and reducing the potential reactivity of these cells [4]. Omalizumab has been used successfully in and is approved for the treatment of disorders in which mast cells are pathogenetically involved such as allergic asthma [5] and chronic urticaria [6]. Therefore, therapy with this antibody in mastocytosis seems worth considering. We present two cases of indolent SM which showed good response to omalizumab treatment.

## 2. Case Presentation

The first patient was a 48-year-old woman with CM for the last 15 years and with diagnosed SM for one year but with diffuse systemic symptoms for the last five years. She also had psoriasis and was formerly diagnosed with cutaneous malignant melanoma and dysplasia of the cervix. SM was verified by one major criterion and three minor WHO criteria: presence of multifocal, dense aggregates of mast cells in bone marrow confirmed by tryptase immunohistochemistry (major criterion) and expression of CD2, CD25, and CD117 in bone marrow, persistently elevated serum tryptase (up to 86 *µ*g/L), and KIT D816B mutation in duodenal and bone marrow biopsies (minor criteria). The patient experienced severe cutaneous itch with widespread rash typical of CM, diarrhoea, palpitations, episodes of musculoskeletal pain, and fatigue daily or almost daily. She was previously treated with oral nonsedating H1-antihistamines at high dose (fexofenadine 180 mg up to four times daily) for six months with reduced occurrence of skin symptoms but not of gastrointestinal or musculoskeletal symptoms. Injections with omalizumab (300 mg s.c. once every four weeks) were initiated, and, within a few months, she experienced resolution of gastrointestinal and cutaneous symptoms, whereas musculoskeletal pain remained. She was followed up for eleven months and continued having a good response of omalizumab. She experienced no side effects of the treatment.

The second patient was a 57-year-old man with CM for the last 30 years and with diagnosed SM for three years. He was otherwise healthy but was on permanent disability pension due to symptoms of mastocytosis. SM was verified by one major criterion and two minor WHO criteria: presence of multifocal, dense aggregates of mast cells in bone marrow confirmed by tryptase immunohistochemistry and with expression of CD117 but not CD2 (major criterion) and persistently elevated serum tryptase (>200 *µ*g/L) and KIT D816B mutation (minor criteria). The patient experienced diarrhoea (loose stools up to five times daily) and severe daily cutaneous itch and widespread cutaneous mastocytosis. He was treated unsuccessfully with PUVA (three times per week) plus high dose of nonsedating H1-antihistamines (ceterizine/levocetirizine 5–10 mg up to three times daily) for two months and, hereafter, with UVA-1 (three times per week) plus high dose of nonsedating H1-antihistamines (ceterizine/levocetirizine 5–10 mg up to three times daily) and montelukast for one year and four months. He was subsequently switched to a strong topical corticosteroid (used two times per day) for one month with no effect. Finally, omalizumab treatment (300 mg s.c. once every four weeks) was initiated but paused for six months after only one injection, as he was unable to attend scheduled ambulatory visits due to a motor vehicle accident and a fractured leg. Treatment with omalizumab was resumed and, after three months, he experienced marked reduction in cutaneous itching as well as resolution of gastrointestinal symptoms. He was followed up for 15 months on omalizumab and continued having a good response with no side effects of the treatment.

## 3. Discussion

Omalizumab inhibits the binding of IgE to the surface of mast cells and basophils by forming complexes with free IgE in serum. This results in downregulation of FC*ε*RI expression on mast cells and basophils and reduction of mast cell and basophil activation [4]. The mechanisms whereby omalizumab decreases symptoms in patients with mastocytosis are not well known. Also, little is known about the pathogenesis of symptoms in mastocytosis patients. Yet, it is plausible that the reduction in mast cell activity caused by omalizumab may reduce these symptoms caused by an excessive number of mast cells.

There are several case reports suggesting that omalizumab may decrease symptoms of mastocytosis, both in its systemic and cutaneous form. To our knowledge, six previous studies with a total of seven patients have reported successful treatment of SM with omalizumab [7–12]. These studies of SM all reported less anaphylactic episodes, with the exception of one patient in whom fewer gastrointestinal symptoms were reported. Another three studies totalling three patients have reported successful treatment of CM with omalizumab [13–15]. These reports all found reduction in skin symptoms (Table 1). In line with our patients, previous studies reported very few side effects of omalizumab, the only exception being Jandus et al. [16] who describe a patient with SM and Hymenoptera venom allergy who was administered omalizumab as add-on therapy to improve venom immunotherapy tolerability and who—during omalizumab treatment—developed sleep disturbances.

We did not taper the omalizumab dose or change the dosing intervals during treatment nor did we monitor IgE or other serological markers among our patients. It is possible that symptom resolution in SM can be obtained with lower doses of omalizumab (e.g., 150 mg) and longer dosing intervals (e.g., every four to eight weeks). Also, it is possible that our patients would have benefitted symptomatically from treatment with oral cromolyn. However, we did not use this drug.

Our report of reduced gastrointestinal and cutaneous symptoms in two patients with SM treated with omalizumab expands on previous findings providing additional insight into the effects of omalizumab for SM.

## Figures and Tables

**Table 1:** Studies of mastocytosis treated with omalizumab.

Study	Patients (*n*)	Dose	Effect
Systemic mastocytosis			
Carter et al., 2007 [7]	2	300 mg/4 weeks	Patient I: no further anaphylactic episodes
			Patient II: reduced gastrointestinal and anaphylactic episodes
Pitt et al., 2010 [8]	1	300 mg/4 weeks	No further anaphylactic episodes
Kontou-Fili et al., 2010 [9]	1	300 mg/4 weeks	No further anaphylactic episodes
Douglass et al., 2010 [10]	1	150 mg/4 weeks	No further anaphylactic episodes or cardiac arrest
Paraskevopoulos et al., 2013 [11]	1	300 mg/4 weeks	No further anaphylactic episodes
Kibsgaard et al., 2014 [12]	1	300 mg/4 weeks	No further anaphylactic episodes
Cutaneous mastocytosis			
Siebenhaar et al., 2007 [13]	1	150 mg/4 weeks	Reduced cutaneous symptoms
Matito et al., 2013 [14]	1	450 mg/4 weeks	Reduced cutaneous symptoms
Sokol et al., 2014 [15]	1	375 mg/2 weeks	Reduced cutaneous symptoms
